# Unaltered Perception of Suprathreshold Contrast in Early Glaucoma Despite Sensitivity Loss

**DOI:** 10.1167/iovs.61.8.23

**Published:** 2020-07-17

**Authors:** Habiba A. Bham, Simon D. Dewsbery, Jonathan Denniss

**Affiliations:** 1 School of Optometry & Vision Science, University of Bradford, Bradford, United Kingdom; 2 Ophthalmology Department, Leeds Teaching Hospitals NHS Trust, Leeds, United Kingdom

**Keywords:** glaucoma, contrast perception, contrast matching, contrast constancy, suprathreshold

## Abstract

**Purpose:**

Glaucoma raises contrast detection thresholds, but our natural visual environment is dominated by high contrast that may remain suprathreshold in early to moderate glaucoma. This study investigates the effect of glaucoma on the apparent contrast of visible stimuli.

**Methods:**

Twenty participants with glaucoma with partial visual field defects (mean age, 72 ± 7 years) and 20 age‐similar healthy controls (mean age, 70 ± 7 years) took part. Contrast detection thresholds for Gabor stimuli (SD, 0.75°) of four spatial frequencies (0.5, 1.0, 2.0, and 4.0 c/deg) were first measured at 10° eccentricity, both within and outside of visual field defects for participants with glaucoma. Subsequently, the contrast of a central Gabor was matched to that of a peripheral Gabor with contrast fixed at two times or four times the detection threshold. Data were analyzed by linear mixed modelling.

**Results:**

Compared with controls, detection thresholds for participants with glaucoma were raised by 0.05 ± 0.025 (Michelson units, ± SE; *P* = 0.12) and by 0.141 ± 0.026 (*P* < 0.001) outside and within visual field defects, respectively. For reference stimuli at two times the detection contrast, matched contrast ratios (matched/reference contrast) were 0.16 ± 0.039 (*P* < 0.001) higher outside compared with within visual field defects in participants with glaucoma. Matched contrast ratios within visual field defects were similar to controls (mean 0.033 ± 0.066 lower; *P* = 0.87). For reference stimuli at four times the detection contrast, matched contrast ratios were similar across all three groups (*P* = 0.58). Spatial frequency had a minimal effect on matched contrast ratios.

**Conclusions:**

Despite decreased contrast sensitivity, people with glaucoma perceive the contrast of visible suprathreshold stimuli similarly to healthy controls. These results suggest possible compensation for sensitivity loss in the visual system.

Glaucoma is characterized by the degeneration and death of retinal ganglion cells leading to irreversible sight loss. Many people with early or moderate stage glaucoma do not experience symptoms and as a result may not seek treatment. The lack of symptoms in the early stages of glaucoma is one reason why many cases of glaucoma remain undetected, and why many people with glaucoma already have advanced vision loss upon initial presentation in clinic.^1^

The effect of glaucoma on the everyday visual experience of those with the disease is not well-understood. This incomplete understanding hampers efforts to produce realistic depictions of scenes as they would appear to a person with glaucoma, as well as to explain the likely visual symptoms to those at risk. Previous studies have used interviews, questionnaires, and forced-choice image selection experiments to further understand the perceptual changes experienced by glaucoma patients.^2^^,^^3^ These studies have found that people with glaucoma perceive increased blur^2^^,^^3^ and increased glare,^3^ and feel that they need more light.^3^ Unlike common depictions of scene perception in glaucoma, patients do not perceive “tunnels” or black patches in their vision.^2^^,^^3^ Despite these studies providing some insight, our understanding of how glaucoma affects patients’ visual perception remains limited.

Current clinical vision tests for glaucoma typically measure contrast detection thresholds or the detection of fixed contrast stimuli across the visual field (static automated perimetry). However, scenes from our everyday visual environment predominantly contain suprathreshold contrast. In the healthy visual system, the effect of stimulus properties on the appearance of suprathreshold contrast differs substantially from that on contrast detection thresholds. The minimum contrast required to detect a stimulus (contrast detection threshold) depends on the spatial frequency content and eccentricity of that stimulus. For instance, greater contrast is required to detect stimuli of high or low spatial frequency, compared with stimuli of medium spatial frequency.^4^ Further, a stimulus presented centrally can be detected at lower contrast than the same stimulus presented peripherally.^4^^,^^5^ However, the apparent contrast of a visible, suprathreshold stimulus is perceived veridically and independently of both spatial frequency and eccentricity, a phenomenon termed “contrast constancy.”^6^^–^^9^ This term was first introduced by Georgeson and Sullivan (1975) in their study comparing perception across spatial frequencies, but may be extended to incorporate the consistent perception of suprathreshold contrast across other properties that affect detection thresholds. Several candidate mechanisms have been proposed to mediate this difference between threshold and suprathreshold vision, including alterations to the contrast gain of spatial frequency-specific channels^6^^,^^10^ and differences in the influence of neural noise under different conditions.^7^

Although decreases in contrast sensitivity in glaucoma are well-known (e.g.^11^^–^^13^), the effects of the disease on the appearance of suprathreshold contrast remain unknown. Retinal ganglion cells play a role in contrast processing and contrast adaptation through alterations to their response gain,^14^^,^^15^ and changes to contrast gain and adaptation have been demonstrated previously in glaucoma.^16^^–^^18^ Understanding how these changes impact the appearance of suprathreshold contrast may help to understand the visual experience of people with glaucoma, potentially leading to improved public information and rehabilitation. Alternatively, if contrast constancy is maintained in glaucoma, this knowledge may lead to an improved understanding of possible compensatory mechanisms for decreased sensitivity in the visual system, and provide evidence of another contributory factor for the lack of symptoms in early to moderate glaucoma.

In this study, we explore the effects of glaucoma on the perception of the contrast of visible suprathreshold stimuli both inside and outside of regions of visual field defect as measured by static automated perimetry.

## Methods

### Participants

Twenty participants with glaucoma (mean age ± SD, 72 ± 7 years) and 20 individually age-matched healthy control participants (70 ± 7 years) participated in the study. Participants were recruited through advertisements in local hospital ophthalmology departments, patient charities, newspapers, and community groups.

All participants had visual acuity better than 6/9.5 (Snellen) in the tested eye and refractive error no greater than 6.00 DS or 3.00 DC. Participants had no ocular or systemic condition known to affect vision except mild cataract (no more than NC3 NO3 C2 P2 on the Lens Opacities Classification System III grading scale^19^) and glaucoma for the participants with glaucoma. Control participants had normal findings on examination of eye health prior to testing. Eye health assessment included perimetry (SITA Standard 24-2, Humphrey Field Analyzer III, Carl Zeiss Meditec, Jena, Germany), Goldmann applanation tonometry (intraocular pressure ≤ 21 mm Hg and difference between the eyes ≤ 3 mm Hg for the control group), slit lamp biomicroscopy, and indirect fundoscopy. We defined a “visual field defect” as a cluster of three or more adjacent points with a pattern deviation *P* of less than 5% and at least one of which is a *P* value of less than 1% based on criteria given by Anderson and Patella.^20^ Control participants were included in the study if perimetry showed no visual field defect and glaucoma hemifield test analysis was within normal limits.

Only participants with glaucoma with a partial visual field defect were included in the study; at least one quadrant of the visual field plot had a visual field defect as defined earlier while at least one of the three other quadrants was without a visual field defect by the definition. Additionally, participants with glaucoma had at least one sector of the retinal nerve fiber layer with thickness outside normal limits (*P* < 5%) on an optical coherence tomography circumpapillary scan (Spectralis; Heidelberg Engineering GmbH). Confirmation of glaucoma diagnosis was obtained from the latest ophthalmology clinic report and/or a reliable history from the patient with evidence of current treatment. If both eyes fit the criteria for the glaucoma group, the tested eye was chosen at random. The tested eye for the control group was chosen at random.

Participants with glaucoma were tested in two spatial locations at 10° eccentricity in two of the four ordinal directions. One test location was chosen in a quadrant with a visual field defect, and one test location was chosen in a quadrant without a visual field defect. Control participants were tested in a single location at 10° eccentricity such that the location tested corresponded to that of their individually age-matched glaucoma participant within an area of a visual field defect. For example, if the glaucoma observer was tested in the right eye superior-nasal quadrant, the age-matched control participant was tested in either the right or left eye in the superior nasal quadrant. Supplementary Figure S1 shows each glaucoma participant's visual field and tested locations.

All participants provided written informed consent in accordance with the tenets of the Declaration of Helsinki before participating in the study. The study was approved by a National Health Service ethics committee. An inconvenience allowance was provided to participants.

### Apparatus and Stimuli

Gabor stimuli (SD 0.75°, random orientation each trial, phase cycling at 1 Hz) with spatial frequencies of 0.5, 1.0, 2.0, and 4.0 c/deg were used. Stimuli were generated in MATLAB 8.5.0 (R2015a; The MathWorks, Natick, MA) using Psychtoolbox (V3.0.14).^21^^–^^23^ Stimuli were presented on a 14-bit calibrated display system (resolution 1920 × 1080, refresh rate 120Hz; Display++, Cambridge Research Systems Ltd, Kent, UK). The mean luminance of the screen was 52.8 cd/m². Appropriate refractive correction was provided with wide aperture trial lenses for the viewing distance of 100 cm that was maintained using a chin and forehead rest. Monocular testing was performed with occlusion of the nontested eye.

Fixation was monitored by eye tracking (LiveTrack FM, Cambridge Research Systems Ltd) with a recording rate of 60 Hz. Central fixation was defined as viewing within a 2.5° radius of the fixation marker/center of the Gabor stimulus. Peripheral stimuli were only presented while central fixation was reported by the eye tracker. Those participants who could not be monitored using the eye tracker (3 participants with glaucoma and 4 controls) were observed using live video monitored by the researcher. In these cases, eye tracking failed owing to small pupils and/or small palpebral apertures.

### Procedure

#### Contrast Detection Thresholds

**Figure 1.**
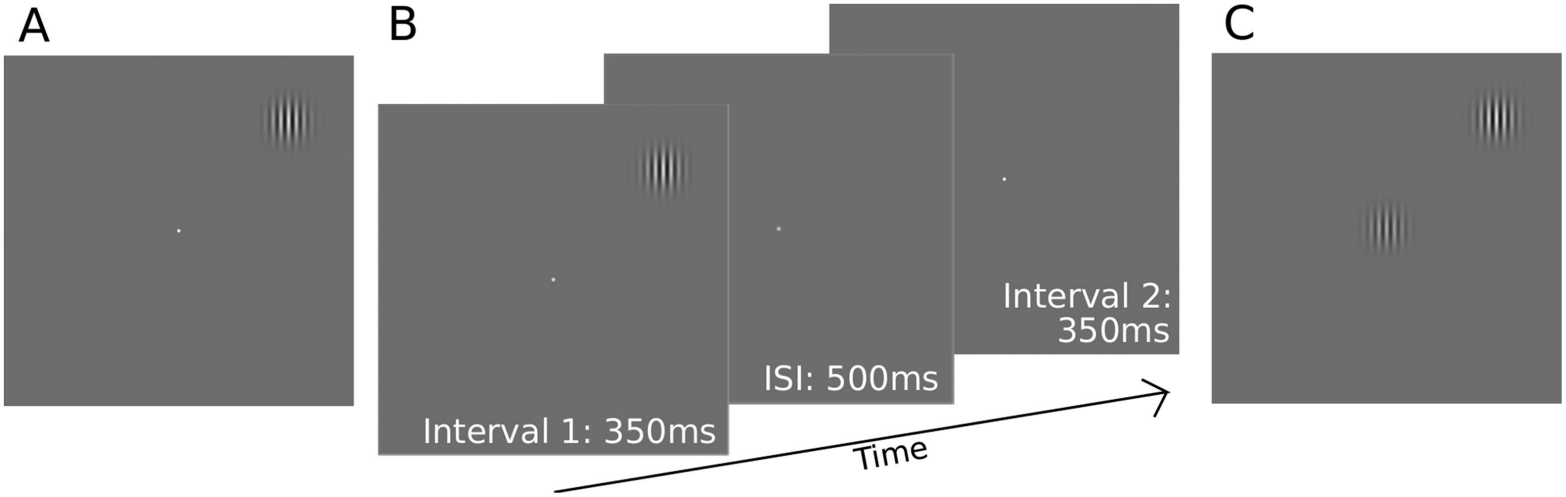
Schematic of the experimental procedures. (**A**) Initial estimates of contrast sensitivity were made by adjusting the contrast of a Gabor stimulus at 10° eccentricity until it was “just visible”, while fixating a central target. These estimates were then used as a starting point in a two-interval forced choice procedure used to obtain final contrast detection thresholds (**B**). The Gabor stimulus appeared randomly in either of the two intervals at 10° eccentricity. Participants indicated in which interval the Gabor appeared by a key press. (**C**) Schematic of the contrast matching task. The contrast of the central Gabor stimulus was adjusted by the participant to achieve a perceptual match with the contrast of the midperipheral reference Gabor stimulus, which was fixed at either two times or four times the detection threshold contrast. The reference stimulus was presented at 10° eccentricity in one of the four ordinal directions. ISI, interstimulus interval.

Contrast detection thresholds for the Gabor stimuli were measured using a two-step process. First, approximate thresholds were obtained using the method of adjustment. Participants focused on a central fixation target and a Gabor stimulus was presented at 10° eccentricity in the ordinal direction in the specified quadrant (Fig. 1A). The participant adjusted the contrast of the stimulus using a dial (CB7, Cambridge Research Systems) until they could “just see it.” One full rotation of the dial clockwise or anticlockwise resulted in a 10% increase or decrease in contrast, respectively. These contrast detection threshold estimates were used as a starting point for the subsequent two interval forced-choice procedure used to obtain final contrast detection thresholds. Stimulus contrast throughout the study was defined using Michelson contrast: (*L*_max_ – *L*_min_)/(*L*_max_ + *L*_min_), where *L*_max_ and *L*_min_ are the maximum and minimum luminance of the stimulus, respectively. Possible contrasts, therefore, range from 0 to 1.

The final contrast detection thresholds were measured using a two-interval forced choice procedure (Fig. 1B). Observers were asked to fixate on the central white spot target; if the observer fixated outside the central 5° diameter of the target, the eye tracker would alert by a buzzing sound and peripheral stimuli were not presented until the observer refixated centrally. Stimuli appeared in one randomly chosen interval for 350 ms, with contrast ramped on and off according to a raised cosine temporal profile, separated by a 500-ms interstimulus interval. Participants identified whether the stimulus appeared in interval one or two by key press. Stimulus contrast was adjusted according to a three down one up staircase procedure, with independent staircases randomly interleaved for each spatial frequency. Stimulus contrast was adjusted by 20% before the first reversal and 10% thereafter. Staircases terminated after six reversals, with the mean of the last four reversals taken as the contrast detection threshold. Contrast sensitivity was calculated as the reciprocal of contrast detection threshold.

#### Suprathreshold Contrast Matching

Suprathreshold apparent contrast was measured for each Gabor stimulus in a matching paradigm. Reference contrast levels were set at two times and four times the detection thresholds obtained prior, as described elsewhere in this article. The reference Gabor stimulus was presented in the midperipheral location at 10° eccentricity, whereas a random contrast stimulus, identical in all other respects, was shown centrally (Fig. 1C). Participants adjusted the contrast of the central Gabor using a dial (method of adjustment) until its apparent contrast matched the peripheral reference Gabor, indicating a match by pressing a button. This process was repeated 12 times in a block for each stimulus condition and the mean contrast matched was taken as the measurement of apparent contrast of the peripheral reference stimulus. To account for differences in contrast detection thresholds between participants, matched contrast ratios were calculated as matched contrast/reference contrast.

A total of eight stimulus conditions were tested for control participants; four spatial frequencies (0.5, 1.0, 2.0, and 4.0 c/deg) and two reference contrast levels for each spatial frequency (two times and four times the detection threshold). For participants with glaucoma, these eight conditions were repeated in two locations, within and outside of a visual field defect. The number of conditions tested was restricted for some participants depending on the initial detection thresholds; if contrast detection thresholds for a particular spatial frequency or test location were greater than 0.25, that condition could not be tested because the reference contrast levels would exceed 100% contrast. The contrast matching task for all testable stimulus conditions was completed in a predetermined randomised order. Fixation was monitored via the eye tracker; if participants fixated outside the central 5° diameter, a black crosshair would appear on the screen surrounding the central Gabor stimulus and the peripheral reference Gabor would disappear. The peripheral stimulus would only reappear once the participant had refixated correctly.

### Statistical Analysis

Statistical analysis was conducted in R version 3.6.1 using the lme4 and emmeans packages.^24^^–^^26^ Because data were collected from two spatial locations in the participants with glaucoma, data were not independent between test locations (within/outside visual field defects) within the glaucoma group. Data were therefore analyzed by linear mixed modelling, accounting for within-subject effects. Six separate linear mixed models were used to test each main effect individually: fixed effects of “group” (three “groups” defined as participants with glaucoma tested within a visual field defect, participants with glaucoma outside the visual field defect, and control participants) and spatial frequency on contrast detection thresholds and contrast match ratios at reference contrasts two times and four times detection thresholds. Random effects of participant were included in each model to account for within-participant effects arising from the participants with glaucoma appearing in two of the three groups. As such, models took the form:

$$y∼1+x+\left(1|\mathrm{Participant}\right),$$

where *y* represents the outcome measure of detection threshold or matched contrast ratio, *x* represents the fixed effect measure of group or spatial frequency, and 1 represents the intercept, with (1|Participant) denoting random intercepts for individual participants.

Basic models including only intercepts and random effects of participant (i.e., *x* = 0) were compared with models additionally including the fixed-effect parameter in question (*x*) by χ^2^ likelihood ratio test of the nested models. Models were fit to the data by maximum likelihood estimation. If likelihood ratio test results had a *P* value of less than 0.05, effects were separated by group and spatial frequency by Tukey post hoc tests on estimated marginal means, also revealing effect sizes. Between group differences are reported as mean ± SE.

## Results

### Contrast Detection Thresholds

**Figure 2.**
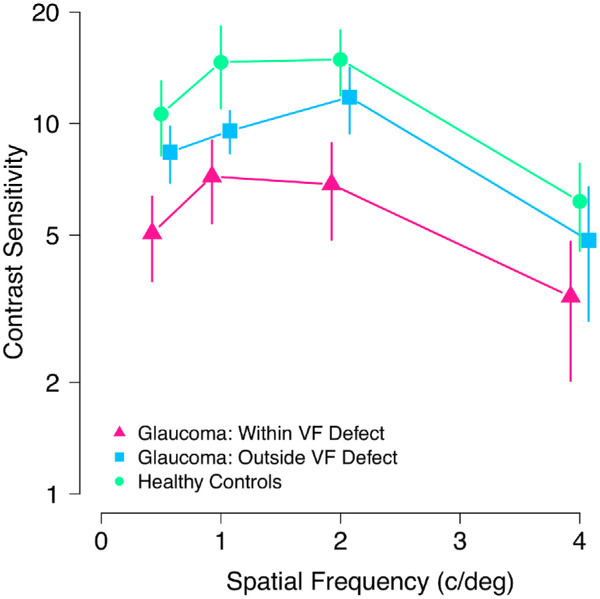
Mean contrast sensitivity for healthy control participants (*green circles*) and participants with glaucoma within their visual field (VF) defect (*pink triangles*) and outside of their visual field defect (*blue squares*). Error bars show 95% confidence interval of the mean.

Detection threshold data are presented as contrast sensitivity functions (contrast sensitivity = 1/contrast detection threshold) in Figure 2. Detection thresholds for the participants with glaucoma overall were raised relative to controls, main effect, χ²(2) = 29.1, *P* < 0.001. Compared with controls, the mean detection thresholds for participants with glaucoma were elevated by 0.141 ± 0.026 (*P* < 0.001) within the visual field defect and by 0.050 ± 0.025 (*P* = 0.12) outside the visual field defect area.

Contrast detection thresholds were spatial frequency dependent, χ²(3) = 89.96, *P* < 0.001. Specifically, thresholds were increased for the 4.0 c/deg stimulus by 0.180 ± 0.024, 0.220 ± 0.024 and 0.221 ± 0.024 compared with 0.5, 1.0, and 2.0 c/deg stimuli, respectively (all *P* < 0.001). Contrast detection thresholds for spatial frequencies of 1 and 2 c/deg were similar (difference in mean detection thresholds, 0.001 ± 0.024, *P* = 1.0), as were detection thresholds for 0.5 c/deg stimuli, 0.040 ± 0.024 (*P* = 0.33) and 0.041 ± 0.024 (*P* = 0.31) higher relative to 1 and 2 c/deg, respectively.

### Suprathreshold Contrast Match Ratios

Three participants with glaucoma were unable to detect the 4 c/deg stimulus at maximum contrast within their visual field defect. A further 13 participants with glaucoma were unable to perform the matching task with reference contrast four times the detection threshold for the 4 c/deg stimulus in the visual field defect area owing to ceiling effects (four times the detection threshold ≥ 1). This left only data from four participants with glaucoma in the reference contrast four times the detection threshold condition for the 4 c/deg stimulus within the visual field defect; this condition was therefore excluded from analysis. To account for ceiling effects among the other conditions, those contrast matching datasets that included more than 4 of 12 matches at the measurement ceiling were removed from the analysis; this applied to 4 control and 6 glaucoma participant datasets in total. One of the removed datasets was from a control participant in the two times detection threshold condition with the 4 c/deg stimulus. All remaining removed datasets were from the four times the detection threshold condition and were distributed, as shown in the Table.

**Table.** Distribution of Datasets Removed From the Four Times the Detection Threshold Reference Contrast Condition of the Contrast Matching Task Owing to Ceiling Effects.

Datasets	0.5 c/deg	1 c/deg	2 c/deg	4 c/deg
Healthy controls	2	0	0	1
Glaucoma within VF defect	1	0	1	–
Glaucoma outside VF defect	1	0	1	2

VF, visual field.

Note that the 4 c/deg condition for the participants with glaucoma within their visual field defect was excluded from analysis entirely.

**Figure 3.**
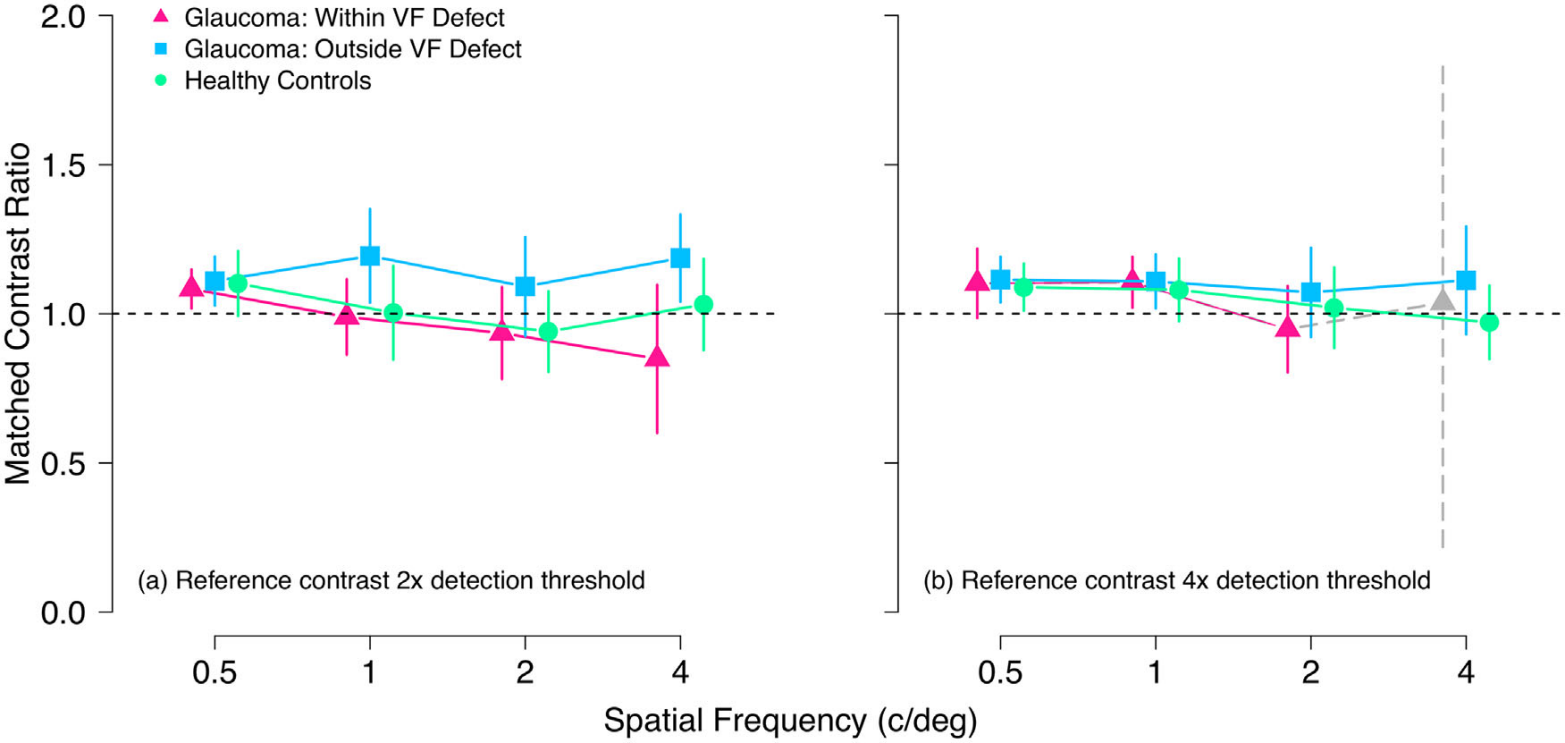
Mean suprathreshold contrast matching functions for reference stimulus contrasts two times (**a**) and four times (**b**) the detection threshold. *Vertical axes* show the ratio between matched and reference contrasts (matched contrast ratio = matched contrast/reference contrast). *Horizontal dashed lines* indicate where matched contrast equals physical contrast (matched contrast ratio = 1.0). Symbols are coded as in Figure 2, except that the *grey triangle* with *dashed error bars* represents the data for the four participants with glaucoma able to complete the 4 c/deg condition at four times the detection threshold. These data were excluded from statistical analysis and are included here for reference only. Error bars show 95% confidence interval of the mean. VF, visual field.

Figure 3 shows group mean contrast match ratios for the two reference contrast levels (two times and four times the detection threshold). For reference stimuli at two times detection threshold contrast (Fig. 3a), there was a main effect of group on contrast match ratios, χ² (2) = 16.4, *P* < 0.001. This effect was caused by a difference between the two tested locations within the participants with glaucoma; matched contrast ratios were 0.16 ± 0.039 (*P* < 0.001) higher outside compared with within visual field defects in glaucoma observers. Contrast match ratios were, however, similar between controls and participants with glaucoma, both within (matched contrast ratios mean 0.033 ± 0.066 lower; *P* = 0.87) and outside (matched contrast ratios mean 0.126 ± 0.066 higher; *P* = 0.14) visual field defects (Fig. 3a).

For the higher contrast reference stimuli at four times the detection contrast (Fig. 3b), matched contrast ratios were similar between control participants and participants with glaucoma in both tested locations (grand mean 1.07 [range, 1.06–1.10]) main effect of group, χ²(2) = 1.1; *P* = 0.58. Contrast match ratios were minimally affected by spatial frequency for both two times- χ²(3) = 6.4; *P* = 0.092, and four times- χ²(2) = 5.9; *P* = 0.054, reference contrasts.

**Figure 4.**
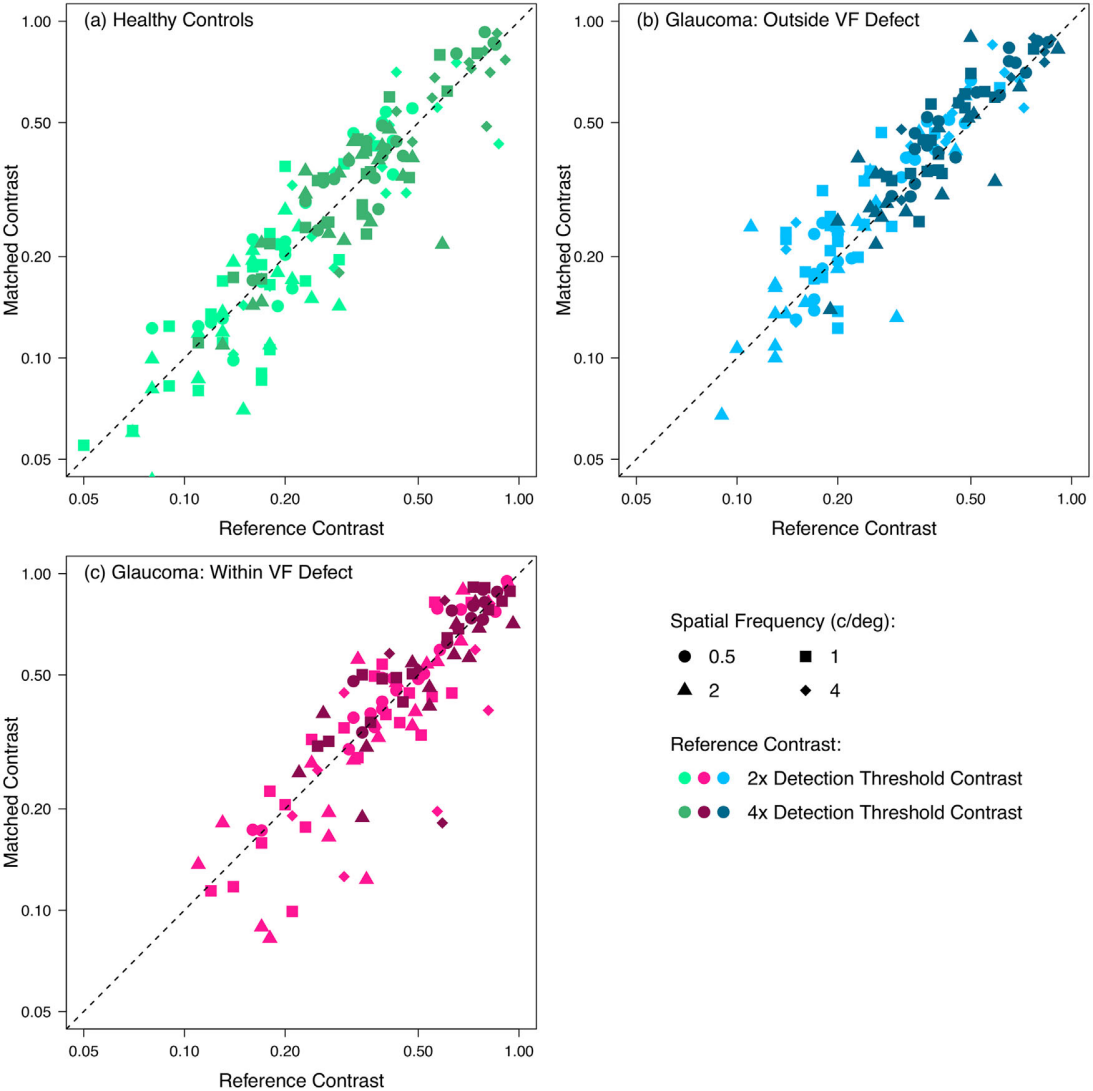
Contrast matches for individual participants in each experimental condition. Data are shown for (**a**) healthy control participants, (**b**) participants with glaucoma outside their visual field (VF) defect, and (**c**) participants with glaucoma within their visual field defect. *Dashed 1:1 lines* indicate perceived contrast matching physical contrast. *Lighter plotting symbols* indicate reference contrasts of two times the detection threshold, and *darker plotting symbols* indicate reference contrasts of four times the detection threshold.

Figure 4 shows individual participants’ contrast matches in each experimental condition. The elevation of detection thresholds in the data for participants with glaucoma can be seen as a relative sparsity of data in the bottom left corner of the plots compared with controls. For all conditions, the majority of points lie close to the diagonal, indicating a perceptual match between the foveal and peripheral locations.

## Discussion

This study aimed to investigate the effects of glaucoma on the perception of the contrast of visible, suprathreshold stimuli. Consistent with previous studies, contrast detection thresholds were increased in the glaucoma group within visual field defects relative to age-similar controls.^13^^,^^17^ However, the perception of suprathreshold contrast was similar between the control and glaucoma groups, particularly in the more suprathreshold higher reference contrast condition. This unaltered perception of suprathreshold stimulus contrast for participants with glaucoma was present both within and outside of visual field defects as measured by perimetry. These results provide further evidence that common depictions of what glaucoma patients see, such as “black tunnel” effects and grayed-out regions, do not accurately represent the perception of scenes by people with glaucoma.^2^^,^^3^ Further, the unaltered perception of suprathreshold contrast may be a factor in the lack of symptoms experienced by many people with early glaucoma, despite significant sensitivity loss measurable by perimetry.

Our finding of spatial frequency independent, near-veridical perception of suprathreshold contrast in healthy vision is consistent with previous literature where it has been termed “contrast constancy.”^6^^–^^10^^,^^27^ Although the neural mechanisms underpinning contrast constancy are undetermined, a number of mechanisms have been hypothesized.^6^^,^^7^^,^^10^ First, it has been widely proposed that a number of independent channels tuned to different spatial frequencies exist within the visual system to deconstruct and interpret the image.^4^^,^^28^^–^^30^ Georgeson and Sullivan proposed that changes in contrast gain within these channels under suprathreshold conditions could compensate for the attenuation in sensitivity to high and low spatial frequencies at threshold, thus equalizing the visual system's response to suprathreshold stimuli of varying spatial frequencies.^6^ Swanson et al.^10^ extended this concept further by developing a model that could predict contrast matching data from contrast thresholds using a small number of medium bandwidth mechanisms tuned to differing spatial frequencies. The model demonstrated that the visual system's response to varying spatial frequencies could be normalized by adjusting the slope of the contrast transfer function (contrast gain) of individual mechanisms within the model.^10^ Brady and Field,^7^ however, proposed an alternative model that assumes contrast gain remains constant across spatial frequency channels under suprathreshold viewing conditions. Brady and Field suggested that contrast constancy is a result of the visual system's response to the signal alone rather than detection thresholds that are affected by the signal to noise ratio.^7^ Brady and Field proposed that higher spatial frequency channels respond to noise more than mid-range spatial frequency channels, resulting in a reduced signal-to-noise ratio and increasing detection thresholds for high spatial frequency stimuli.^7^ However, their empirical data show contrast constancy as soon as stimuli are suprathreshold, which is inconsistent with other literature showing a gradual flattening of contrast matching functions with increasing suprathreshold contrast.^6^^,^^10^^,^^27^^,^^31^

The results of this study suggest that the mechanisms underlying contrast constancy in the healthy visual system may be intact in glaucoma and able to compensate for pathologic loss of sensitivity. Alternatively, or additionally, further mechanisms may aid compensation for sensitivity loss. It is possible that loss of sensitivity may be accompanied by decreased perceptual surround suppression via alterations to the gain and/or inhibition of downstream visual mechanisms. This may enable an overall perceptual response broadly similar to the predisease state to be maintained despite the decreased sensory input and, combined with existing contrast constancy mechanisms, may be one possible explanation for the present findings. A recent study has investigated two measures of lateral inhibition in the relatively intact central visual field of people with advanced glaucoma, finding no difference from healthy controls.^32^ One of their measures, the difference in log contrast sensitivity between 1 and 4 c/deg can also be tested in our data. On this measure we also found no differences between any of the groups (*P* = 0.92, linear mixed model), suggesting that there is no change in lateral inhibition between glaucoma within or outside visual field defects and healthy participants. Further research is needed to explore the mechanisms underlying suprathreshold contrast perception in glaucoma.

The results of this study are consistent with previous studies investigating suprathreshold contrast perception in other disorders of the visual system, including amblyopia^9^ and nystagmus.^33^ In people with atrophic AMD, exudative AMD, and juvenile macular degeneration, Mei et al.^31^ found that, despite a flattening of the contrast matching functions, there was still a significant difference in contrast matching data between controls and those with maculopathy, although not as large as the difference between detection thresholds. This finding may be explained by not testing participants with maculopathy sufficiently far above threshold to reach contrast constancy; the highest contrast tested was 0.56, and all observers were assessed at the same contrast levels despite the maculopathy group having increased detection thresholds, relative to controls.^31^

Because our everyday visual environment is dominated by suprathreshold contrast, the findings of this study provide some insight into the everyday visual experience of people with glaucoma. However, there are several reasons why our results should be interpreted with caution when considering everyday vision. First, participants were tested monocularly; thus, we are unable to comment on the effects of binocular interactions or the compensation for visual field defects in one eye by relatively intact corresponding visual field in the fellow eye. Further assessment of vision in glaucoma under binocular viewing conditions would be valuable in furthering our understanding of the daily visual experience of those with glaucoma. Second, we used simple Gabor stimuli to enable precise control of stimulus parameters, such as spatial frequency, contrast, and eccentricity. However, findings using these stimuli may not accurately reflect vision under complex natural viewing conditions. Studies have shown that the visual system responds differently to complex stimuli and natural scenes compared with simple stimuli,^34^ so further work investigating apparent contrast in natural scenes in glaucoma may be valuable. Finally, a further potential limitation of the present study is that the contrast-matching paradigm used assumes that participants’ central vision was normal, but we did not measure foveal contrast sensitivity directly using the Gabor stimulus. Some studies have shown changes to central vision in early glaucoma.^35^ Changes to contrast perception in central vision cannot explain our results, however, because apparent contrast of stimuli both within and outside of visual field defects, where contrast detection thresholds were markedly different, was close to veridical (Figs. 3b and 4). Decreased apparent contrast of the central stimulus, if present, could only explain the contrast matches in one, but not both, visual field regions.

The results of this study do not imply that people with glaucoma do not experience visual impairment. Whatever the mechanism of the unaltered suprathreshold contrast perception observed herein, there is no mechanism that could compensate for a total loss of retinal input. Thus, when all retinal ganglion cells signaling a region of visual field are lost, that area becomes blind. Related, we were unable to test most participants with glaucoma within their visual field defect at four times the detection threshold reference contrast with the 4 c/deg stimulus. This was because detection thresholds for this stimulus were elevated beyond 25% contrast; thus, the appropriate reference contrast (>100%) could not be produced. A contrast detection threshold of 25% in this study was approximately four times the mean normal contrast detection threshold for the medium spatial frequencies. In clinical perimetry, a detection threshold four times higher than normal equates to a loss of 6 dB. Although the detection thresholds measured in this study are not directly comparable with perimetric thresholds owing to differences in the stimulus and its presentation, it is clear that many more advanced glaucomatous visual field defects would cause contrast detection thresholds to be increased beyond 25% contrast. Therefore, although our results are consistent with early glaucoma being asymptomatic, they are also compatible with more advanced glaucoma causing visual impairment.

This study has demonstrated that people with glaucoma perceive the contrast of visible, suprathreshold Gabor stimuli similarly to age-similar healthy observers despite decreased contrast sensitivity. This finding is consistent both within and outside of clinically measured visual field defects. The results suggest active or passive compensation for reduced sensory input in the damaged visual system that normalizes responses to suprathreshold contrast, possibly similarly to the mechanisms of contrast constancy in normal vision. The results also provide further evidence for the inaccuracy of common depictions of vision with glaucoma that show black or gray areas obscuring scenes. Further research is required to explore these mechanisms and to better understand the daily perceptual experience of people with glaucoma.

## Supplementary Material

Supplement 1
