# Supplementary material for: Unaltered Perception of Suprathreshold Contrast in Early Glaucoma Despite Sensitivity Loss
Source: Invest Ophthalmol Vis Sci. 2020 Jul 17;61(8):23. doi: 10.1167/iovs.61.8.23 (PMC7425759; doi:10.1167/iovs.61.8.23)
Supplement: Supplement 1 [file iovs-61-8-23_s001.pdf]

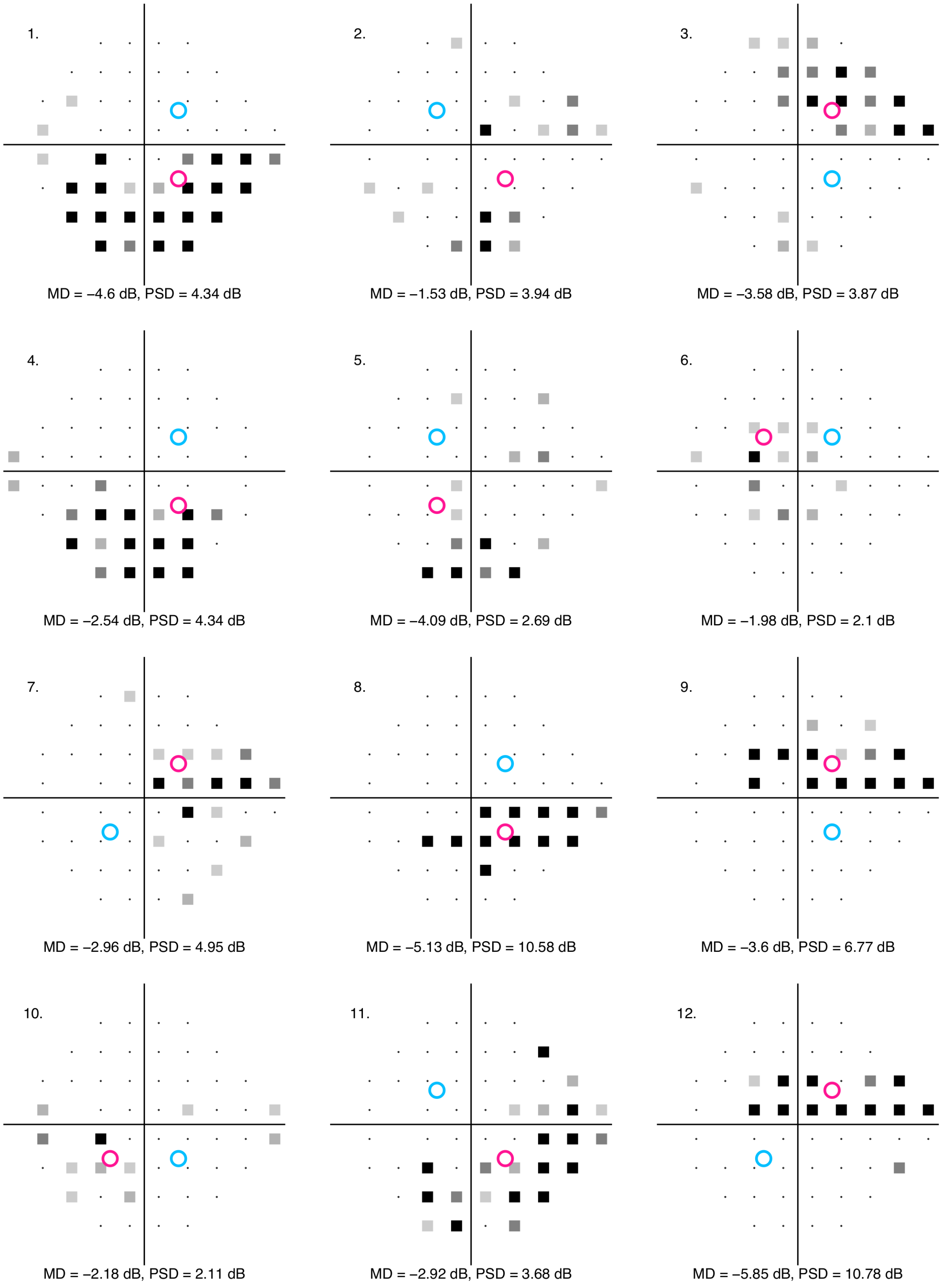
1.
MD = −4.6 dB, PSD = 4.34 dB
2.
MD = −1.53 dB, PSD = 3.94 dB
3.
MD = −3.58 dB, PSD = 3.87 dB
4.
MD = −2.54 dB, PSD = 4.34 dB
5.
MD = −4.09 dB, PSD = 2.69 dB
6.
MD = −1.98 dB, PSD = 2.1 dB
7.
MD = −2.96 dB, PSD = 4.95 dB
8.
MD = −5.13 dB, PSD = 10.58 dB
9.
MD = −3.6 dB, PSD = 6.77 dB
10.
MD = −2.18 dB, PSD = 2.11 dB
11.
MD = −2.92 dB, PSD = 3.68 dB
12.
MD = −5.85 dB, PSD = 10.78 dB

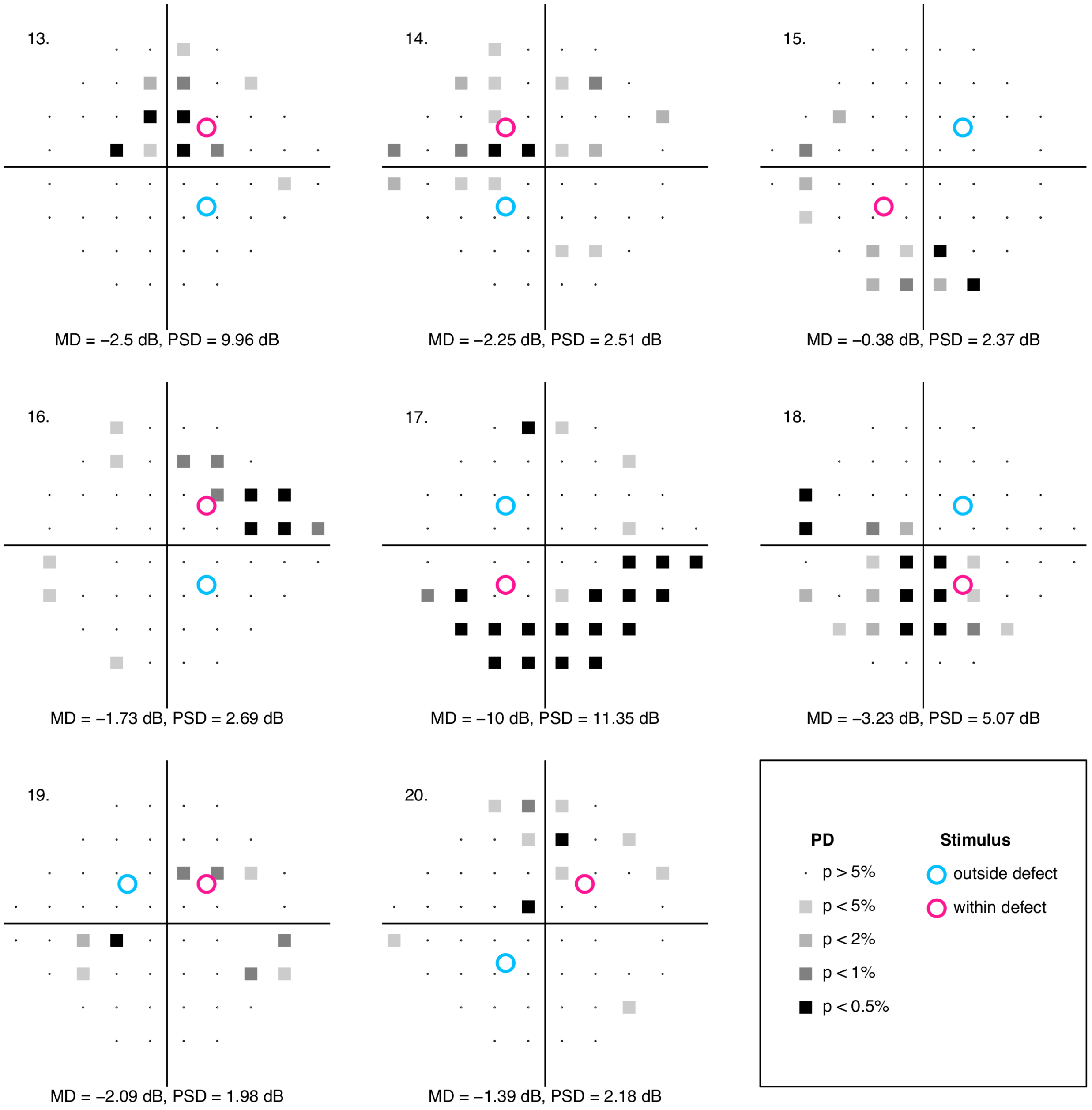

Supplementary Figure: Visual field pattern deviation plots for each glaucoma participant and the test locations used. See key to symbols in the bottom right panel. Red circles indicate the test location within the defect, blue circles indicate the test location outside the defect. Red/blue circles are shown to scale corresponding to the area containing 95% of the Gabor stimulus energy. Visual field test points are not drawn to scale. MD =Mean Deviation, PSD =Pattern Standard Deviation.
